# Aortic wrapping is life-saving in high-risk acute aortic dissection and intramural haematoma

**DOI:** 10.1093/icvts/ivac254

**Published:** 2022-10-07

**Authors:** Thierry Carrel, Juri Sromicki, Martin Schmiady, Raed Aser, Ahmed Ouda, Paul Robert Vogt

**Affiliations:** Department of Cardiac Surgery, Clinic for Cardiac Surgery, University Hospital, Zürich, Switzerland; Department of Cardiac Surgery, Clinic for Cardiac Surgery, University Hospital, Zürich, Switzerland; Department of Cardiac Surgery, Clinic for Cardiac Surgery, University Hospital, Zürich, Switzerland; Department of Cardiac Surgery, Clinic for Cardiac Surgery, University Hospital, Zürich, Switzerland; Department of Cardiac Surgery, Clinic for Cardiac Surgery, University Hospital, Zürich, Switzerland; Department of Cardiac Surgery, Clinic for Cardiac Surgery, University Hospital, Zürich, Switzerland

**Keywords:** Aortic dissection, External wrapping, Surgery, High risk, Early outcome

## Abstract

Aortic wrapping is a controversial repair in patients presenting with acute type A aortic dissection or intramural haematoma, but this method may be a potential alternative to medical treatment or conventional repair in patients aged >80 years and in those presenting with prohibitive co-morbidities such as stroke, circulatory collapse, full oral anticoagulation with the last generation drugs. We report on 5 high-risk and/or patients over 80 years who received external aortic wrapping with or without cardiopulmonary bypass during the last 18 months. All survived the procedure and could be extubated early postoperatively. No patient remained on the intensive care longer than 2 days and all were discharged without additional complications. Postoperative radiological control was acceptable and no patient had any new aortic event up to 18 months postoperatively.

Against the currently accepted strategy, we found external aortic wrapping an interesting alternative method to stabilize the ascending aorta and prevent delayed rupture in the setting of acute aortic dissection and intramural haematoma that would qualify for operative repair. We believe that wrapping should be discussed in patients that would be otherwise denied for conventional aortic repair.

Acute aortic dissection and intramural haematoma (IMH) are 2 of those aortic conditions that fall under the moniker acute aortic syndrome along with penetrating aortic ulcer and iatrogenic or traumatic aortic injury. IMH is often considered an early stage of acute aortic dissection (AAD) that requires the same treatment. However, in some instances (aortic diameter ≤45–50 mm, haematoma ≤10 mm and absence of significant hemopericardium and aortic insufficiency), conservative treatment with blood pressure control and monitoring through repeated imaging may be justified, especially in older and high-risk patients [1].

The prognosis of patients presenting with acute aortic dissection depends on the promptness of diagnosis and appropriate treatment. Immediate medical treatment includes analgesia with opioids that beneficially influence anxiety and respiratory distress and anti-impulse therapy with strict control of blood pressure using beta-blockers or calcium antagonists. Emergency surgery is the gold standard in most patients with type A acute aortic dissection [2]. Decision-making around surgical treatment includes how much proximal and distal aortic repair is needed as well as a strategy for body perfusion and organ protection (e.g. cardiopulmonary bypass, hypothermia and method of cerebral perfusion).

A primary entry tear in the ascending aorta is addressed by replacement of the ascending aorta using an interposition prosthetic graft with the proximal anastomosis performed at the level of the sinotubular junction and a limited replacement of the concavity of the aortic arch with a circular open arch distal anastomosis.

This technique is particularly indicated in case of normally functioning aortic valve without significant aortic root enlargement. It is the most expedient procedure and is sufficient in most cases, although a limited initial approach may result in additional procedures later in life [2, 3].

Recently, some articles concerning the revival of an old technique, that is called aortic wrapping, have been published and critically commented [4–8]. This technique had been described >40 years ago to minimize the surgical approach in high-risk patients with ascending aortic aneurysm [9]. In the recent period, we have observed an increasing proportion of patients referred because of acute aortic dissection and intramural haematoma, probably due to the unrestricted availability of cross-sectional imaging all over the country. As a consequence, a substantial number of the referred patients are rather old (>80 years) and/or present with multiple and severe comorbidities and other high-risk situations such as stroke and coma, post-resuscitation with unclear neurology and concomitant coronary artery disease with severely reduced left-ventricular function, while others are under long-term current NOAC intake [10]. These factors contribute to a challenging decision-making for immediate surgery.

## CLINICAL CASES

Based on some recent reports with encouraging results, we have performed external wrapping in 5 patients presenting with acute type A aortic dissection (4 cases) and IMH (1 case). The main non-aortic comorbidity was: severe stroke in 1, acute corona virus disease 2019 (COVID-19) disease with a recent history of thromboembolic disease and new oral anticoagulation in 1 and a recent history of interventional approach to multivessel coronary artery disease with a double antiplatelet therapy in 1; 3 patients were 84, 85 and 88 of age and this was considered main ‘risk factor’.

**Figure 1:**
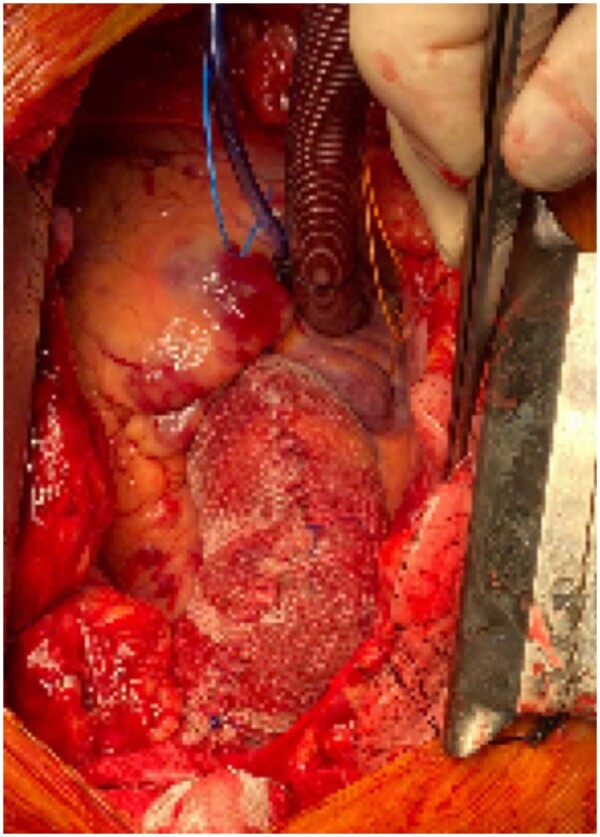
Intraoperative view of external wrapping with a polypropylene mesh graft and a reduction of the overall diameter by 5–10 mm.

These 5 patients received a most complete wrapping using either a vascular prosthetic graft or a polypropylene mesh around the ascending aorta with proximal epicardial, respectively, distal adventitial sutures for a durable fixation to prevent further dislocation (Fig. 1).

**Figure 2:**
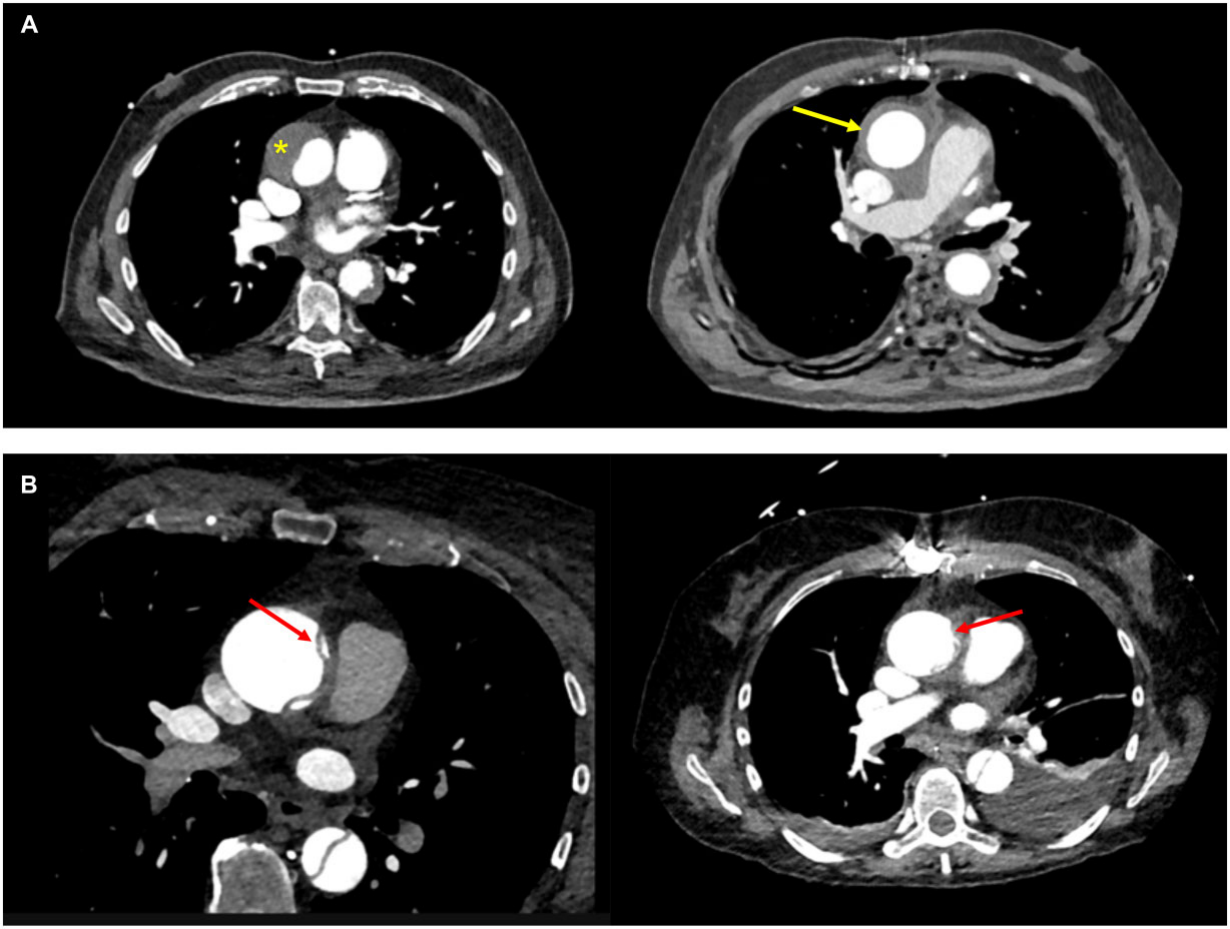
(**A**) Preoperative CT scan of the 87-year-old patient with an intramural haematoma of the ascending aorta (left) with a thickness of 22 mm (*) and postoperative control 3 months following wrapping (right): the intramural haematoma has practically completely disappeared (arrow) and the aortic diameter has remained stable. Retrospectively, it was suspected that the primary tear occurred in the proximal descending aorta and extended in a retrograde way up to the ascending aorta. (**B**) Preoperative CT scan in a 72-year-old female under oral anticoagulation demonstrating acute aortic dissection with the flap in the ascending aorta (left with red arrow) and postoperative control 12 months later (right) showing an almost disappearance of the dissecting membrane in the ascending aorta (red arrow) and absence of aortic growth, peri-aortic haematoma and/or pericardial effusion.

In 3 patients, the procedure was performed under normothermic cardiopulmonary bypass on beating heart and in 2 patients in whom the circular preparation of the ascending aorta could be easily performed, the operation was performed off-pump with the circuit filled in the operating room in case of technical troubles. The median duration of the procedure was 2 h and 30 min (from 2 to 3 h 20 min). All patients survived the procedure, and there was no need for blood transfusion in any of these patients that all could leave the intensive care unit within 48 h. The median duration of hospitalization was 10 days; no patient developed postoperative pericardial effusion requiring drainage. The follow-up extends from 3 to 18 months. No late death nor any aortic-related event has occurred so far. Computed tomography (CT) scan showed a complete regression of the IMH and a thrombosed and shrinked false channel following dissection in 1 case each. No further increase in aortic diameter was observed in the 4 patients who presented with acute type A dissection but complete thrombosis of the false lumen in 1 of them (Fig. 2A and B).

## COMMENT

Aortic wrapping has been proposed decades ago to less invasively treat moderate aortic enlargement in higher-risk patients with moderate enlargement of the ascending aortic diameter [9]. In the presence of acute aortic dissection and IMH, some series have reported promising results concerning early- and mid-term survival in selected patients [4–7] while postoperative CT scan surprisingly showed complete regression of the false aortic lumen in a substantial number of patients. Despite these results, these series have been discussed controversially [8].

Older and higher-risk patients with larger IMH and/or acute type A aortic dissection with the primary intimal tear in the aortic arch are probably the best candidates for this less-invasive type of repair. As correctly stated by the majority of groups that are not favourable to aortic wrapping, the manipulations around the ascending aorta in the setting of acute dissection are very critical, especially when there is a large haematoma between the aorta and the pulmonary artery [5, 7]. In these cases, beating heart cardiopulmonary bypass may be a good option together with a strict control of blood pressure. Results following aortic wrapping have been reported to be favourable in well-selected patients [4–7]. Suematsu *et al.* [4] reported a technique of stepwise wrapping in 49 patients with IMH and major comorbidities for whom conventional surgery was considered high risk with a considerable potential for unfavourable outcome. They reported no hospital mortality and a very low incidence of postoperative complications. The same author reported this technique in 43 patients presenting with acute aortic dissection with 1 hospital death (2.3%) and a low complication rate of 4.6% for neurologic disorder and renal failure [5]. The follow-up survival rate was 95.3% and 91% at 1 and 3 years. There was no aortic-related death during follow-up. At 1 year after surgery, complete remodelling of the ascending aorta was obtained in 30 patients (85.7%). A French group reported a series of 35 patients who received aortic wrapping in the setting of acute aortic dissection with a 30-day mortality of 9% [6].

Aortic wrapping will never take a major place in the treatment of patients presenting with acute type A aortic dissection; however, it is a less-invasive option that may allow early- and mid-term survival in very old patients and in those who may suffer from another life-limiting disease. This option should also be discussed before definitive denial of a patient for surgery. In these patients, aortic wrapping may be attractive because it allows a more expedient recovery. Shortage of intensive care resources, as it has been observed during the COVID-19 pandemic, may also be a valuable reason to proceed with such a procedure. In fact, no patient remained longer than 48 h on the intensive care unit (ICU) in this series, despite the fact that 3 of them were between 84 and 88 years of age. We were surprised by the uncomplicated recovery observed in these high-risk patients and had to revise our negative initial opinion about this technique all the more since no patient presented any further complication in a follow-up of up to 18 months.

## LIMITATIONS AND CONCLUSION

There are of course limitations for such a report: small number of patients and shorter follow-up of 18 months for the longest observation. Furthermore, the decision to proceed with wrapping was left at the discretion of the operating surgeon. However, randomized studies in the setting of acute aortic dissection are very unlikely to be performed in the future.

Nevertheless, we consider aortic wrapping as a reasonable alternative to conventional repair in high-risk or elderly patients. The procedure allowed shorter operation time and intensive care duration in these 5 patients, and the midterm follow-up was very satisfactory with no aorta-related events. But this technique still needs further validation by experienced aortic teams.

Future directions: it is likely that the number of referred old and very old patients suffering from acute type A aortic dissection or IMH will further increase due to the demographic changes. With the potential for restricted resources in the future, simpler and more expedient procedures like aortic wrapping may become welcome and better accepted in the cardiosurgical community, especially for marginal patients when the decision to attempt a rescue procedure is supported by the patient and his relatives in contrast to a full palliative approach with a very limited survival [11].

## Funding

No funding in relation with this work is reported.

**Conflict of interest:** none declared.

## Data Availability

The data underlying this article will be shared on reasonable request to the corresponding author. Interactive CardioVascular and Thoracic Surgery thanks the anonymous reviewer(s) for their contribution to the peer review process of this article.

## References

[1] Sorber R , Hicks CW. Diagnosis and management of acute aortic syndromes: dissection, penetrating aortic ulcer, and intramural hematoma. Curr Cardiol Rep 2022;24:209–16. 35029783 10.1007/s11886-022-01642-3 PMC9834910

[2] Malaisrie SC , Szeto WY, Halas M, Girardi LN, Coselli JS, Sundt T et al; AATS Clinical Practice Standards Committee: Adult Cardiac Surgery. 2021 The American Association for Thoracic Surgery expert consensus document: surgical treatment of acute type A aortic dissection. J Thorac Cardiovasc Surg 2021;162:735–758.e2. 34112502 10.1016/j.jtcvs.2021.04.053

[3] Benedetto U , Dimagli A, Kaura A, Sinha S, Mariscalco G, Krasopoulos G et al Determinants of outcomes following surgery for type A acute aortic dissection: the UK National Adult Cardiac Surgical Audit. Eur Heart J 2021;43:44–52. 34468733 10.1093/eurheartj/ehab586 PMC8720141

[4] Suematsu Y , Inoue T, Nishi S, Kurahashi K, Arima D, Yoshimoto A. Stepwise external wrapping procedure for type A intramural hematoma. J Thorac Cardiovasc Surg 2022;164:31–8.e1. 32977967 10.1016/j.jtcvs.2020.08.025

[5] Suematsu Y , Inoue T, Nishi S, Kurahashi K, Yoshimoto A. Aortic remodeling after stepwise external wrapping for type A acute aortic dissection. Ann Thorac Surg 2022. 10.1016/j.athoracsur.2022.05.070. 35863391

[6] Guihaire J , Ramadan R, Nottin R. Off-pump wrapping for acute type A dissection: alternate option in patients deemed inoperable. Ann Thorac Surg 2022. https://doi.org/10.1016/j.athoracsur.2022.01.051. 10.1016/j.athoracsur.2022.01.051 PMC9676564 35189116

[7] Vento V , Multon S, Ramadan R, Deleuze P, Fabre D, Guihaire J et al Outcomes of urgent aortic wrapping for acute type A aortic dissection. J Thorac Cardiovasc Surg 2020. 10.1016/j.jtcvs.2020.10.136. 33419559

[8] Nienaber CA , Hoschtitzky A, Yuan X. Commentary: external wrapping in proximal intramural hematoma of the aorta: what's behind the magic? J Thorac Cardiovasc Surg 2022;164:40–1. 33023748 10.1016/j.jtcvs.2020.09.023

[9] Robicsek F , Daugherty HK, Mullen DC. External grafting of aortic aneurysms. J Thorac Cardiovasc Surg 1971;61:131–4. 5540453

[10] Sromicki J , Van Hemelrijck M, Schmiady MO, Krüger B, Morjan M, Bettex D et al Prior intake of new oral anticoagulants adversely affects outcome following surgery for acute type A aortic dissection. Interact CardioVasc Thorac Surg 2022;35(1):ivac037. 10.1093/icvts/ivac037. PMC9252133 35258082

[11] Wang A , Montgomery D, Brinster DR, Gilon DR, Upchurch GR, Gleason TG et al Predicting in-hospital survival in acute type A aortic dissection medically treated. J Am Coll Cardiol 2020;75:1360–1. 32192665 10.1016/j.jacc.2020.01.015

